# The self-perceived role of tech champions in municipal healthcare services—a descriptive qualitative study

**DOI:** 10.1186/s12913-025-12994-1

**Published:** 2025-07-01

**Authors:** Sissel Pettersen, Hilde Eide, Anita Berg

**Affiliations:** 1 Faculty of Nursing and Health Sciences, Nord University, P.O. Box 474, Namsos, N-7801 Norway; 2 Centre for Health and Technology, Faculty of Health Sciences, University of South-Eastern Norway, PO Box 7053, Drammen, N-3007 Norway

**Keywords:** Tech champion, Municipal health care service, Technology, Health professionals, Role performance, Implementation, Qualitative research

## Abstract

**Background:**

Health professionals performing tech champion roles have been identified as key personnel for successful technology adaptation. However, studies of tech champions’ roles in small organizations and from their own perspective are limited. This study explores how health professionals perform tech champions roles in municipal healthcare services.

**Methods:**

This study is based on eight semi-structured interviews with health professionals holding tech champion roles in municipal healthcare services. Purposeful sampling from five locations in three Norwegian municipalities was conducted to ensure diversity in technologies and contexts. Braun and Clarke’s [2022] description of reflexive thematic analysis guided the data analysis.

**Results:**

We found that tech champions hold an undefined role, holding neither predefined job descriptions nor assigned clear-cut dedicated tasks. Performance of the tech champion role appears to be highly contextual. Tech champions must be able to perform a set of sub-roles simultaneously and customize their role performance to the respective stages of the implementation processes, specific technologies, and the implementation contexts. Thus, their enthusiasm, professional and technological competencies, and organizational know-how provided them with credibility and influence to fulfill their perceived mission of promoting, adopting, and supporting the use of technologies in the municipal healthcare service.

**Conclusion:**

The tech champion’s role is undefined by management, but the champions themselves hold a clear understanding of their tasks and sub-roles in the technology implementation process. Tech champions need to hold several professional, technological, and personal competencies, as well as organizational know-how, to fulfill their perceived mission of supporting technology implementation in their services. The findings indicate that while it is important who holds the tech champion role, tech champions are not strategically integrated into municipal health technology implementation processes.

**Supplementary Information:**

The online version contains supplementary material available at 10.1186/s12913-025-12994-1.

## Background

Healthcare systems globally have long sought to take advantage of technological innovations via digitalization [1]. In Norway, enthusiasm for such technology is based on several parallel changes affecting municipal healthcare services, including the task shift from specialized hospital care to municipal health services [2], an ageing population [3], increasing numbers of patients with complex healthcare needs [4, 5], and the global shortage of health personnel affecting Norway [6]. This shift calls for initiatives to implement healthcare technologies and address how municipal healthcare services should be organized and delivered. The implementation of healthcare technology is challenging traditional work processes in the respective services and depends on the skills of patients, management, and health personnel, the usability of the technology, and the context [7, 8].

Successful implementation of technology is vital to ensure the quality and sustainable development of healthcare services and may help make health services more efficient and precise [9]. Implementing technologies in healthcare service will change how healthcare personnel work [10], and healthcare personnel need competence and knowledge, education, practice, and support to help patients operate these new technologies [11].

Technology could help health personnel organize their day by using programs that optimize capacity and thereby strengthen their collaboration and communication. Technological tools could also aid health personnel in decision making in their daily work, increasing patients’ security and ensuring the quality of healthcare services, e.g., greater precision and efficacy and using less time to acquire necessary knowledge [12].

Not all health personnel and patients will be receptive to technology. Use of technology could also arouse fear among health professionals of being replaced [13], and problems could arise as healthcare personnel and IT professionals understand technology from different perspectives [14]. Further, technology also affects patient and staff privacy, raising ethical issues [15]. All these factors must be addressed by organizations implementing technologies within their services.

According to the Norwegian national eHealth strategy [16] there is a need to clarify roles and the division of responsibility in implementing technologies, including the role and responsibilities of champions. A champion could be an expert on technology and local workflow that assists their peers in adopting and using the technology [17] or someone highly supportive of technology with the ability to engage others [18].

Health professionals performing champion roles have been identified as key personnel for successful technology adaptation [19, 20]. A champion is just one of several factors in the successful implementation of technology in municipal healthcare services [21]. Hendy and Barlow [22] remind us that while having a champion does not mean success, the tech champion approach to other health personnel is of immense importance. Recent reviews have revealed modest knowledge of the champions’ role, performance, tasks, and activities in implementing innovations in healthcare generally [23] and specifically in facilitating technology [24, 25]. Studies including champions have often focused on large-scale implementation processes with champions as one of several measures, reporting the effects of the presence or absence of champions [23]. Some studies state that a champion encourages, inspires, and engages other health personnel to use technology [17, 26–28], while one study is clear that the champion role must be voluntary instead of designated [29]. The voluntary champion acts proactively, explaining and sharing information, while a designated champion would speak neutrally about the technology, giving only practical and limited information [29]. Rigby et al. [2023] calls for knowledge of skills for the role, while Santos et al. [23] urges more detailed descriptions of what strategies tech champions use when implementing technology. This may be due to the diversity of health service organizations with countless technologies, which in turn may be why it is not possible to prepare a defined role description that suits all. Furthermore, there is a call for dedicated studies of the champion role, e.g., Hall et al. [30] and Shea [20] and for studies from the perspective of health professionals performing the role of technology implementation champions in small organizations [17].

Thus, there is a lack of knowledge on this issue from the perspective of health personnel holding these roles themselves [23–25]. Prior studies have revealed that health personnel in champion positions may hold a variety of titles, e.g., superuser, facilitator, and IT clinician [31–33], and positions, e.g., physician champions, clinical champions, and telemedicine champions [34–36]. In a review of health personnel in the champion role in 2024 we identified that 15 of the 23 included studies used the term champion in different combinations [25]. Based on the commonalities in the role related to champion technology, this study uses the term “tech champion.”

Tech champions could serve as one of the measures of a complex implementation process. Implementation science is often used to describe, understand, and evaluate implementation processes using theory, models, and frameworks [37]. The implementation process describes the steps to make technology work, often involving a planning phase, training phase, going-live phase, and full implementation phase, e.g., Fixsen’s model of the implementation process [38]. Other models focus specifically on the determinants of successful implementation or the social processes of technology adaptation, e.g., Normalization Process Theory [39]. This study of tech champions’ perceived role performance is contextualized within implementation processes, which thus are not included as a part of the study objective per se. The aim of this study was to explore how health professionals perform tech champions roles in municipal healthcare services.

## Methods

The study has an exploratory qualitative interview design with an underlying inductive approach. A qualitative study design was considered appropriate due to limited insights into the self-perceived performance of health personnel in tech champion roles. Purposeful sampling [40] was used to choose participants who could give rich information about performing the tech champion role. Semi-structured interviews for this study were conducted to explore and describe how champions perceive and perform their role to gain an understanding of how the implementation process is supported from their perspective. According to Hamilton and Finley [41], a small sample is useful for understanding how things happened and why, allowing us to gain an in-depth understanding of the implementation process with a smaller sample of techfunding champions. The study follows the Norwegian Law of Ethics and National Guidelines of Research Ethics [42]. The collection and storage of personal data were considered and approved by the Norwegian Agency for Shared Services in Education and Research [43] (reference number 403183). The study follows the Consolidated Criteria for Reporting Qualitative Research (COREQ) developed by Tong et al. [44]; Additional file 1).

### Study context

The study was conducted in municipal primary healthcare services in three municipalities of Norway. These healthcare services are publicly funded and provide several types of services for the inhabitants, e.g., short-term services such as rehabilitation and home nursing care, and long-term care in patients’ homes, nursing homes, and sheltered housing for persons with disabilities [45]. The 357 municipalities in Norway (January 1, 2024) vary in size, ranging from 215 (Utsira) to 717,710 (Oslo) inhabitants [46].

The first municipality had between 20,000 and 25,000 inhabitants, the second between 5,000 and 10,000 inhabitants, and the third between 1,000 and 1,500 inhabitants. Our study was conducted from April 2023 to October 2023 in various primary healthcare services. For an overview of the various types of health care services in the study, please refer to Table 1. The healthcare services included in this study have worked with technology implementation for several years and have applied several technologies. At the time of the study, the health professionals in tech champion roles had hold their roles for 1–5 years.

### Study settings and recruitment

To explore how health personnel perform tech champion roles in municipal healthcare services, the recruitment was performed in several steps guided by a criterion-based purposeful sampling procedure. This involves identifying and selecting individuals or groups who are especially knowledgeable about or experienced with a phenomenon of interest [47]. First, we identified potential municipalities with health professionals in the roles of tech champions within their services using Internet searches and through a regional network for technology implementation. We recruited from municipalities of varied sizes and areas of work. Second, we relied upon the head of healthcare services in each municipality to anchor the study, help us with recruitment as a gatekeeper, and provide contact information to department managers. Gatekeepers play an essential role in the generation of good research data by holding key roles within an organization, through which they can provide access to participants with specific characteristics [48]. The gatekeepers sent our request to managers, who provided the tech champions with the study information sheet and contact information. Those interested in participating in an interview volunteered by using the contact details in the information sheet via a digital registration link or the email address. To be included, the health professionals needed to have a three-year professional education at the bachelor level and a minimum of one year practicing as a tech champion. Verbal and written consent was obtained from all participants. The concepts of information power and data richness [49] guided the considerations regarding the number of participants included in the study. Due to the limited number of participants recruited by purposeful sampling, the findings cannot be transferable to a larger population. The participants in the study provide valuable insights, as tech champions’ self-perceived roles are understudied. By holding high methodological transparency, reporting on the recruitment criteria, and providing a thorough description of the study context and participant characteristics [50], the study can lay the ground for future studies.

### Interview guide and data collection

The interview guide included open-ended, probing questions like “What do you perceive as your tasks as a tech champion?,” “What competences are needed to perform the tech champion role?,” and “What are the challenges of performing this role?” (interview guide, Additional file 2). The questions were designed to enable the participants to talk about how they performed the role of tech champion in their respective departments and related to the various technologies they were using. The authors did not establish a relationship with the participants before the study. The interviews were conducted in person by the first author and the participant at the participant’s workplace and lasted for 42–62 min. The first author’s background is as a health professional familiar with implementation processes but has no prior experience within the field of health technology implementation. Field notes were written after the interviews.

The interviews were audio-taped and transcribed verbatim by a professional transcriber. The transcripts and results were not returned to participants for comment or correction, but the first author contacted three participants regarding details of their years of professional practice. None of the participants dropped out of the study.

### Study participants

Eight tech champions from three municipalities participated in the study, holding positions in five different departments/combined workplaces in their municipality. The services involved are as follows: Home nursing means that healthcare personnel go to the home of a person in need of help to provide services. A rehabilitation service consists of an interdisciplinary team of health professionals providing intensive rehabilitation services to people who live at home or in institutions. A nursing home is a healthcare service for those who need 24/7 assistance due to their health condition. In two municipalities, the tech champions worked in combined workplaces for both home nursing and nursing homes, and the respective tasks are distributed based on needs. With one exception, they had no dedicated time to perform their role, but the role was assigned as a non-formalized position on top of their ordinary work. All tech champions were led by a middle manager serving as the head of the department. Of the tech champions, six were nurses, one was a physiotherapist, and one was an occupational therapist. They had between 8 and 21 years of practice and had performed the tech champion role for 1–5 years in three different municipalities. While three tech champions worked with technologies that served patients (e.g., medicine dispensers, alarms, GPS), five worked with technology that could help healthcare personnel in their daily work (electronic health records (EHR), quality improvement systems, e.g., electronic systems that ensure good quality and follow-up of users, register deviations, and document statutory services, and medication management). Further details on the tech champions are shown in Table 1.

**Table 1:** Participant characteristics

Nr	Profession	F/M	Years in practice/as tech champion	Workplace assisted the following service(s)	Technology
1	Nurse	F	14/5	Home nursing	Digital home technology (GPS, sensors, alarms, medicine dispensers, digital monitoring)
2	Nurse	F	13/4	Home nursing	Digital home technology (sensors, digital monitoring, medicine dispensers)
3	Occupational therapist	M	8/3	Rehabilitation service serving all health and care services in the municipality	Digital home technology (medicine dispensers, digital monitoring, sensors, GPS)
4	Nurse	M	18/1	Nursing home	Electronic health record
5	Physiotherapist	M	19/1	Physiotherapy service	Electronic health record
6	Nurse	F	21/4	Nursing home	Quality and patient safety technology (quality system that registers deviations, storage systems, collaboration board)
7	Nurse	F	14/2	Home nursing and nursing home	Medication management technology (closed medicine loop for health personnel)
8	Nurse	F	20/2	Home nursing and nursing home	Medication management technology (closed medicine loop for health personnel)

### Data analysis

**Fig. 1:**
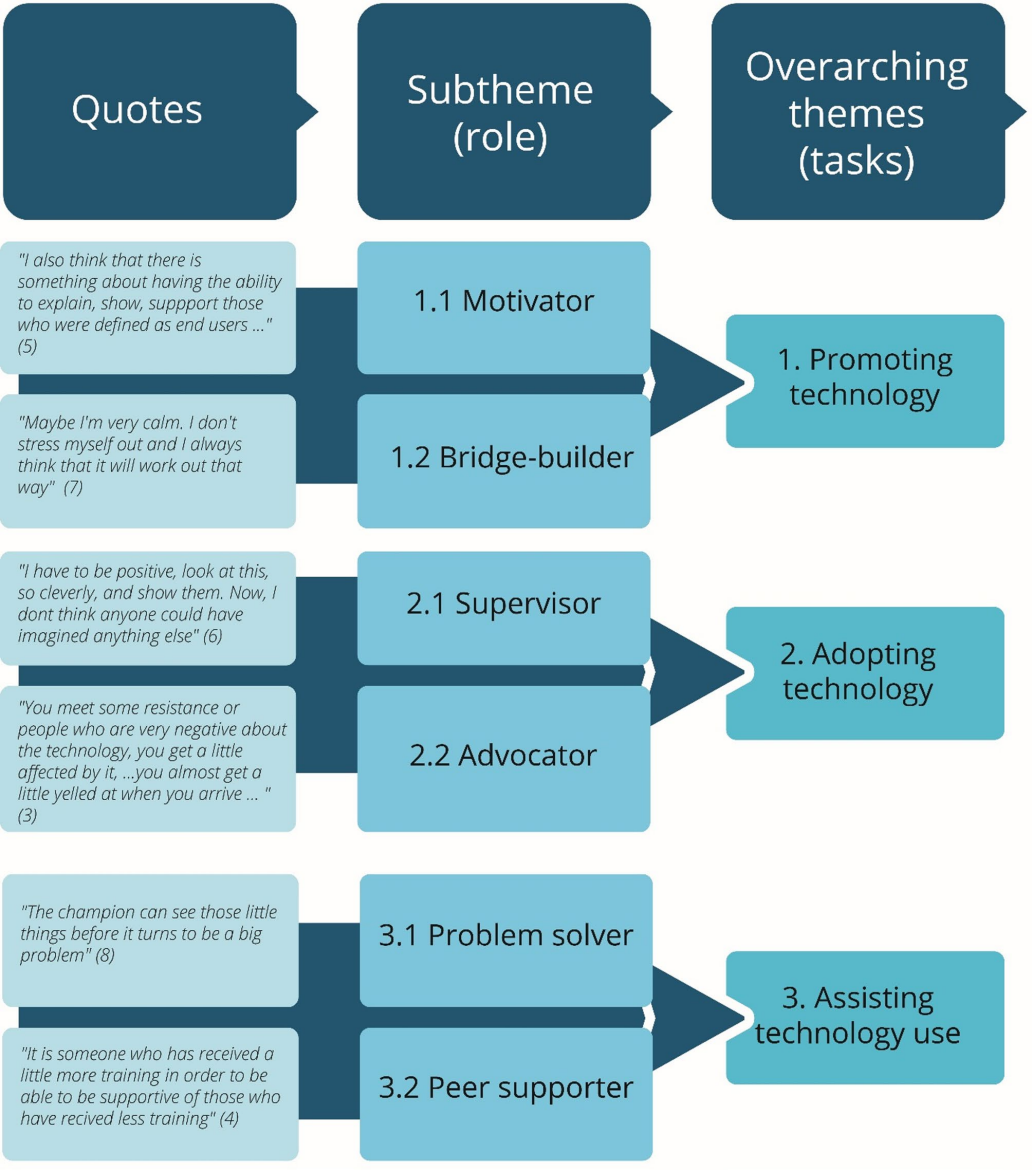
Illustration of coding, and overview of sub-themes and associated overarching themes

Braun and Clarke’s [51] descriptions of reflexive thematic analysis (RTA) guided the data analysis. We used this data-driven approach to explore and interpret patterns across the collected data. An RTA approach is underpinned by reflexive and sensitive practice due to the research question [52] and is not performed as a step-by-step standardized procedure [53]. The first author conducted the initial coding, a common approach in RTA [51], to become familiar with the dataset. An example of the coding is provided in Fig. 1.

To ensure a reflective engagement in the data, the analysis pended between in-depth analyses, searches for patterns across data and contexts, and refinements of themes by the authors. To acknowledge researcher subjectivity, all authors attended several analytic meetings and drew upon each other’s varied expertise and competencies within qualitative interviews, implementation, and health science. The authors hold different health professional backgrounds and have professional experience from serving in various parts of the municipal health care services, while currently holding academic positions. Shared concepts were identified and listed in a written form to give a name for each theme. At the end of the data analysis, we agreed on a description of three overarching themes and six sub-themes. In this phase of the analysis, we realized that some of the themes could relate to the more general tasks in the role, and some of them related more directly to the hands-on role performance. Based on this the themes were reorganized as overarching themes with associated sub-themes.

## Results

In the following, we present the result of health professionals performing tech champion roles in municipal healthcare services under three overarching themes: (1) “Promoting technology,” (2) “Adopting technology,” and (3) “Assisting technology use,” with associated sub-themes (as shown in Fig. 1).

Tech champions seem to know what to do and what was expected of them.

*I feel that I have a lot of freedom and can make decisions on my own*,* I think that’s great. I feel that I’ve been trusted that I do the job I am supposed to*. (Participant 3, occupational therapist, digital home technology)

Having a tech champion role means that you are given the responsibility by your manager to give other health professionals training, assist them, and ensure that everything works as it should. Often it was suppliers that asked for a tech champion; the manager then asked individual health professionals to take the role, or they held a position where it was natural to take on the role. In some cases, health professionals ask for the role themselves, when the manager asks for someone to do so. Four tech champions requested the role, three were assigned the role, and one volunteered upon the request of their workplace. None of the tech champions had received a job description, but they were provided with manuals for the use of the specific technologies. Some of the tech champions had participated in the preparation of routines for the use of specific technologies to tailor them to the local context of implementation.

All tech champions had a special interest and motivation for the technologies, but no one received extra payment for the role. The tech champions had an informal position, but they formed their role based on their working context.

## Promoting technology

Tech champions described their main task as promoting technology. This entailed establishing positive attitudes toward technology implementation and the specific technologies among their peers in their departments and supporting collaboration between the various stakeholders who must work together to realize the potential of the technology. Through this task, they held two complementary roles: motivator and bridge-builder.

### Motivator

Motivating healthcare personnel to use the technology was perceived as vital to the success of its implementation.

*As tech champions*,* we must motivate them to open their [healthcare personnel] eyes. Especially when new ones arrive*,* if there are changes for the patients that I see will be a strain from time to time*,* we could relieve that a bit.* (Participant 1, nurse, digital home technology)

All tech champions said that they were personally interested in using technology and passing their engagement on to other health professionals. Their motivational effort was also connected to anchoring an understanding among the health professionals.

*I must tell them how it works and what we can gain from it and what the purpose of the implementation is. I must be clear about that. It is important that they are involved quite early in the process*,* to enable overall joint understanding for all staff*. (Participant 2, nurse, digital home technology)

Explaining and demonstrating the technology was also perceived as important to nudge staff motivation and build staff confidence to master the technology independently.

*To take the initiative by informing and demonstrating to all employees. That is the key*,* really*. (Participant 3, occupational therapist, digital home technology)

Being a step ahead of their colleagues as a firsthand enthusiast was perceived as imperative to successful implementation. In this stage, they also had to focus on collaboration, both inside and outside the organization.

### Bridge-builder

The bridge-builder role entailed having organizational know-how and establishing connections with relevant collaborative partners and suppliers, as this enables implementation in department practice and ensures necessary support if problems and errors occur.

It was considered imperative to have an overview of who to ask in case of questions, problems, and errors that might arise, which entails having local know-how within the organization and relations with collaborative partners.

*You must have an overview and knowledge of all the departments and all the people who sit around there in the system so that you can collaborate with them. That is also important*. (Participant 3, occupational therapist, digital home technology)

The bridge-builder role required that the tech champion know their organization so as to be able to help and guide their peers if needed. However, the tech champions noted, for example, that it was difficult to use an HER system developed for a context other than municipal healthcare and the suppliers could not always give suitable answers. To resolve this problem, they arranged a visit to learn from another municipality that had implemented the system. As one tech champion stated:

*Before start-up … to talk to someone who uses it [the technology] … Because there is something about talking to those knowing the implementation pros and cons and learning from their firsthand experiences.* (Participant 5, physiotherapist, EHR)

This highlights how the tech champions had to be proactive and prepared to contact collaborative partners, e.g., suppliers, management, administrative staff, and peer tech champions, to provide the healthcare personnel and department the resources and support needed for implementation of the technology in their practice.

## Adopting technology

In implementing technology in the healthcare service, the tech champions described how they had to hold the roles of supervisor and advocate. The interests and knowledge of technology among healthcare personnel varied, particularly between those who had grown up with technology and those who had not, and the staff held both positive and negative attitudes. Regardless of the health professionals’ interest, the tech champions had to make them pull in the same direction to enable adoption of the technology. Customizing supervision was imperative, as well as mediating between the management implementation measures and the staff’s concerns regarding the ethical, legal, professional, and personal points of view. Thus, in adopting technology, the tech champion played the role of supervisor and advocate.

### Supervisor

The tech champions supported both interested and not-so-interested healthcare personnel in implementing technology. Often, they started with general information for all healthcare personnel, followed by the opportunity to ask questions. Further, they provided supervision in hands-on usage of technology in practical situations.

The tech champion used practical situations to ensure that everyone in the healthcare service was familiar with every step of the procedure. When the tech champion was carrying out their job as a health professional, they would invite those who had never performed the different procedures to participate.

*For some [health professionals]*,* the threshold is high. They do not dare to ask in plenary. So*,* I try to be a little more outgoing*,* ask how it is going? … Have you done this before? Before I do it myself … If someone says no*,* then I ask if they want to do it … That is the moment you must act*,* right? … It is in practical situations that you are more receptive to learning when you need to do it yourself*. (Participant 4, nurse, EHR)

Some tech champions used the opportunity to supervise the most interested healthcare personnel first to get allies on board.

*Yes*,* I supervise those who are interested and who I can see can do it*,* who can help me … as far they have access to it. Because not everyone has access to everything I have. And training such mini-tech champions is probably a clever idea too.* (Participant 6, nurse, Quality and patient safety technology)

One solution for those who were skeptical was to offer the option of a trial period before an evaluation. This period is encouraged to adopt the use of technology among both healthcare personnel and patients.

Another way of adopting technology to benefit patients was “to listen in the corridors” to the staff’s perceptions of the patients’ needs and suggest new technologies, e.g., so the patients had more privacy by using GPS or were able to plan their daily routines using a medicine dispenser. Additionally, this technique could be used to gain information about those not using the technology and to offer them individual support.

### Advocator

Not all healthcare personnel were satisfied with the technology, which resulted in the tech champion feeling they had to perform as an advocator. The tech champion felt responsible for adopting and arguing for the technology.

*It is well known that it should be warm hands and not cold technology.* (Participant 3, occupational therapist, digital home technology)

The tech champions were affected by critiques and experienced situations where healthcare personnel almost yelled at them; this resulted in discussions and could be stressful.

*There are some frustrations about technology*,* and I feel like that*,* I must try to calm down*,* even though I feel frustrated myself. That is the most challenging part of the role. I do not feel anyone yells at me personally*,* but it is not always easy to defend management’s decisions … I agree with a lot of their frustrations*,* but it does not help to contribute to it*,* hype up the mood even more*,* nothing good comes of that.* (Participant 4, nurse, EHR)

The tech champion had to highlight the positives of using the technology, determine what the healthcare personnel find difficult, and try to provide a solution. Sometimes the tech champions were confused about who they should represent as an employee, leader, and technology provider.

The tech champions provided supplementary training and allied with others in the healthcare service to help those who struggled. They were sometimes asked by healthcare personnel, especially the oldest ones, if they could perform tasks that included technology use because they seldom used the technology. This mediator role was not always easy, but the tech champions thought that the fact that the technology was decided by the leaders and not by them helped them in this role. One of the tech champions said:

*I try to understand what they [who should use the technology] are wondering about and I strive to not have a top-down approach helping them. It is easy to be perceived as instructive and simplifying when you are a tech champion. You need to hold a sensitive approach because many of them are unsure.* (Participant 4, nurse, EHR)

To retain credibility, one tech champion decided to implement the technology step by step to try to calm down the employees, and sometimes it was necessary to state that they did not have all the answers. The advocator role caused frustration, but by having their own phrases or actions of what to do or say when health professionals reacted in different situations, the tech champions could continue in the role.

## Assisting technology use

To assist in the use of technology, the tech champion helped healthcare personnel solve problems that could not be foreseen and supported their peers. Being able to predict future events based on experience was a characteristic of being able to assist in using the technology. The problem solver and peer supporter roles were prominent when technology became well known within the service. In this phase the tech champion proceeds into a more reactive role, acting when needed.

### Problem solver

By being a tech champion over time, one tech champion described how you could hear the specific noise of the technology and tell the healthcare personnel what was wrong.

*Suddenly*,* a noise came from the medicine cabinet. But I know the sound*,* someone had not put the drawer in place correctly*. (Participant 7, nurse, medication management technology)

Performing the role over time turned the tech champions into experts in technology, and one part of the role of a problem solver was to do things that would not be noticed to avoid bigger problems. However, some problems could not be foreseen.

*We usually act when problems occur*,* and I would take it from there if there was something wrong. They [health professionals] must report any deviations and message me so I know the reason*,* which will make it easier for me to go further. But this does not always happen*,* but then I will find it in the computer system*,* it just takes a little longer.* (Participant 7, nurse, medication management technology)

The above quote indicates that the tech champion acts on demand during this stage. Some things could be solved immediately, but in some cases, depending on the technological problem, troubleshooting requires more effort.

Those tech champions working with the EHR and quality systems used electronic support and handbooks, but this was sometimes not enough. The tech champions at EHR solved this problem by contacting other municipalities working with the same program. The quality program support services were often based on a procurement agreement, which could limit ways of contacting the company other than by a digital support service. This required extra time with the computer to search manuals, for which there was no time in their daily practice, which made it difficult. The tech champions thought there were more problems in the first year after implementation, as healthcare personnel gained more knowledge about using the technology over time.

### Peer supporter

Ordinary support in daily practice was a role for the tech champions, and the tech champions received fewer questions sometime after the technology was implemented. The tech champions felt that they were competent and had an overview of the field and products for which they were responsible. One of the tech champions pinpointed the responsibility for a particular job in healthcare services:

*It sounds like you are supposed to be super good at what you are doing*,* but it is about having a different responsibility for it*. (Participant 8, nurse, medication management technology)

To be responsible for a certain task, e.g., the primary contact for wounds or assistive devices, is an ordinary job in healthcare services, but the tech champion role may require extra work. Part of the role of supporting colleagues was to act before problems occurred so that they could plan vacations and avoid running out of medicine or hygiene items.

The tech champion could not always support the healthcare personnel immediately because of their job in the clinic.

*If there are phone calls while I am in a morning procedure with a patient*,* I must tell them*,* I cannot help you now. This would not give me a bad conscience. I would do it when I could*,* and then I would go and help the others*. (Participant 4, nurse, EHR)

Tech champions had time at meetings to present the technology and gave one-to-one support, especially to those returning from sick leave or maternity leave or to seniors less familiar with and confident in using technology. The supporting role consisted of staying ahead of problems by having an overall view and of being a discussion partner and support when needed.

## Discussion

This study has explored how health personnel perceive their role as tech champions in municipal healthcare services. The study provides firsthand accounts of tech champions who have held the role for several years and across a variety of contexts. Our main findings are that the tech champions held a set of sub-roles simultaneously and customized their role performance to the various stages of the implementation process, the specific technologies, and the implementation context. With one exception, they had no time dedicated to performing their role, but the role was assigned as a non-formalized position on top of their ordinary work. The tech champions thus hold an undefined role, neither holding predefined job descriptions nor being assigned clear-cut dedicated tasks. They are not championing just one, but most often several technologies at the same time in various stages of the implementation process to support the rapidly increasing number of technology-related tasks that the health personnel are expected to take care of. To fulfill their role, the tech champion needs enthusiasm, professional and technological competencies, and organizational know-how to provide them with credibility and influence to fulfill their perceived mission of promoting, adopting, and supporting the use of technologies in the municipal healthcare service.

### Non-linear efforts in technology implementation

**Fig. 2:**
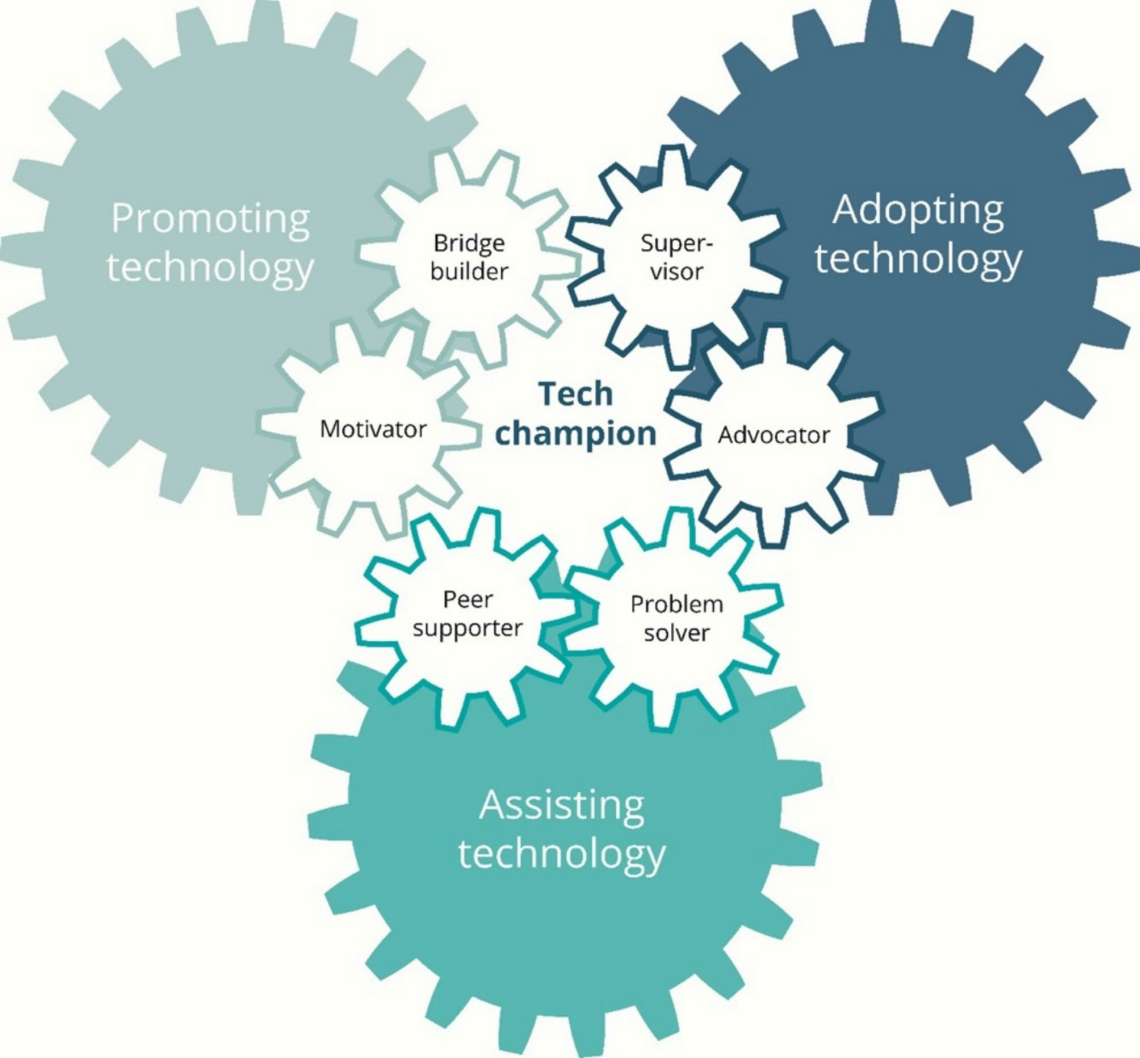
Illustration of the tech champions tasks of promoting, adopting, and assisting technology and the associated sub-roles

Our study found that the tech champion had to customize their role performance to the various stages of the implementation process. The tech champion role appears to be highly contextual and depends on the level of competencies among the staff as well as the facilitation by the management responsible for the implementation. A major finding in our study was that the tech champions had to organize their time continuously between assisting the health personnel, enabling the technology to function in the everyday healthcare delivery in their departments, and performing their ordinary job. The tech champions felt that their main tasks were to aid the promotion and adoption of the technology and to assist in its implementation in their department. However, their efforts did not follow a linear implementation process that successively followed predefined steps as described in the implementation science literature [38]; rather, their tasks to promote, adopt, and assist technology and the attached sub-roles were related to multiple ongoing and intertwined activities. These activities were steered by the arrival of new technologies, troubleshooting problems with the implemented technologies, the turnover, absence, or loss of interest of health personnel, and the perceived expectations of management and suppliers to ensure that the technology functions on a daily basis. The correlation between tasks and the associated sub-roles are illustrated in Fig. 2. This finding aligns more with implementation theories like Normalization Process Theory [39], focusing on the social and the interactional aspects of technology implementation.

The finding relates to the fact that they are not championing just one, but most often several technologies in various stages of implementation to support the rapidly increasing number of technologies related to tasks expected to be taken care of by health personnel. Additionally, these technologies often entail multipurpose components, e.g., EHR consisting of components such as patient journals and e-messages within and between the health services and medication management. This may explain why it is not possible to prepare a defined role-description suited to all tech champions in all municipal health care contexts, and why there is such a variety in terms and lack of clear-cut role descriptions in prior studies [27, 54, 55].

### Tech champions perform multiple roles simultaneously

Our study finds that the tech champions had to be able to perform six sub-roles simultaneously. These roles in turn required that tech champions had the competence to be able to promote, adopt, and assist technology use, which required pedagogical, organizational, and technological knowledge.

A review by Laukka et al. [56] identified seven roles for the leaders of healthcare services implementing health information technology. Several of these role descriptions are like the findings in this study (e.g., our motivator and their supporter, or our peer supporter and their facilitator) or equivalent (e.g., advocator) to the tech champion sub-roles in this study. This may indicate that these roles are important sub-roles for all those who hold facilitating positions in health technology implementation processes. Thus, it may be questioned what the best division of labor may be between health service leaders and tech champions. Further, how can management facilitate the success of health personnel who hold a tech champion role. If the tech champions have no dedicated time, training, or resources to perform their roles, their success is highly dependent on their competence, skills, organizational knowhow, and personal dedication.

The tech champions’ organizational know-how was important to avoid potential problems or fix unforeseen trouble. Knowing the service and the organization well was perceived as crucial to success in technological implementation. This finding is supported by Obwegeser et al. [57] who highlight the need to know the organization to be able to help others. Not only organizational know-how but also knowing the staff in person gave the tech champions credibility and eased their work in establishing trust in the technology among the staff.

One challenge in performing the tech champion role was related to the lack of health technology competencies in their basic training as health professionals. This could be related to the fact that it had been several years since their professional training. While some studies have argued for employing newly graduated nurses as tech champions [58, 59], Gui et al. [17] claim that this is not enough; while one might use a “teenager that knows technology” as an assistant, those who know the organization must do the strategic planning to ensure adaptation to local work contexts. To perform their sub-roles, tech champions had to hold a set of more generic competencies. As highlighted by George et al. [60], general skills, such as motivation and communication, are important attributes of a champion. Summarized, our findings emphasize that tech champions need to hold a set of professional, technological, and pedagogical competencies, as well as organizational know-how.

All the participants had a special interest and motivation to take advantage of technology within their services, and thus were not trained, compensated (in time or payment), or included formally in the implementation. Accordingly, we may ask whether the tech champion was fully equipped to hold this position without having been involved in the decision regarding the choice of technologies and implementation strategies. Our study showed that the tech champion was included after the decision regarding technology implementation and the specific technological solutions had been made by the management in the municipal healthcare service. Combined with the finding of the lack of role descriptions, this may indicate that tech champions are part of the management’s implementation strategy, thus having no formal roles or resources such as time and role-specific training and are not involved in the choice of technologies. Such information is often lacking in studies of technology implementation, where the champion is described as a measure of successful implementation in healthcare services [23, 29]. Based on our findings, we ask whether the responsibility of the tech champion is too extensive and results from a lack of detailed implementation plans and strategies by the management of the municipal healthcare services.

### Study strengths and limitations

This study contributes to the knowledge of tech champions’ perceived role performance in municipal healthcare services by providing firsthand experiences from tech champions in a variety of settings and technologies. Thus, based on a limited number of participants, the study provides novel knowledge that can form a basis and a framework for future studies. This study adds to the body of research providing evidence to support decisions regarding tech champions as a measure of health technology implementation. Some of the tech champions in this study had been recruited by the heads of healthcare services acting as gatekeepers, which could exclude some participants in cases where information about the study was not passed on to the various departments in healthcare services and all those performing the tech champion role.

## Conclusions

Being a tech champion for the implementation of technology requires both technological and professional competencies, as well as organizational knowledge, to enable them to perform a set of sub-roles to fulfill their perceived mission of promoting, adopting and assisting technology in municipal healthcare services. This requires that the management have in-depth knowledge of the health professional’s competence and capacity when choosing a tech champion. By being chosen and responsible for teaching healthcare personnel to use tools in everyday work, tech champions had an impact on how health services were provided or carried out. As the tech champion functions as a hub between the management, suppliers, and staff, the choice of a wrong tech champion decreases in the tech champion’s personal engagement, or their absence is risks to the technology implementation process. Further studies of the performance of tech champion roles on a larger scale and in different contexts could strengthen the knowledge of tech champions as a measure of technology implementation in healthcare services. Dedicated studies exploring the tech champion as a measure from the perspective of leadership could also enlighten the management and organizational aspects of the role in technology implementation processes.

## Supplementary Information

Additional file 1.

Additional file 2.

## Data Availability

The dataset generated and analyzed during the current study are not publicly available because such approval was not given as part of the participants’ consent. Data cannot be shared openly to protect study participant privacy. On reasonable request the corresponding author may consider contacting the participants to request approval for sharing the anonymous dataset. The dataset is in Norwegian language. Contact sissel.pettersen@nord.no.

## Abbreviations

EHR: Electronic Health Record
RTA: Reflexive Thematic Analysis
WHO: World Health Organization
SIKT: Norwegian Agency for Shared Services in Education and Research
