# Supplementary material for: The self-perceived role of tech champions in municipal healthcare services—a descriptive qualitative study
Source: BMC Health Serv Res. 2025 Jul 1;25:856. doi: 10.1186/s12913-025-12994-1 (PMC12220061; doi:10.1186/s12913-025-12994-1)
Supplement: Supplementary file 1 — Additional file 1. [file 12913_2025_12994_MOESM1_ESM.docx]

**Consolidated criteria for reporting qualitative research (COREQ): a 32-item checklist for interviews and focus groups**

Developed from: Tong A., Sainsbury P., Craig J. Consolidated criteria for reporting qualitative research (COREQ): a 32-item checklist for interviews and focus groups, IJQHC 2007;19(6),349–357. <https://doi.org/10.1093/intqhc/mzm042>

| **Topic** | **Item no.** | **Guide questions/description** | **Reported on page no.** |
| --- | --- | --- | --- |
| **Domain 1: Research team**  **and reﬂexivity** | | | |
| ***Personal Characteristics*** |  |  |  |
| Interviewer/facilitator | 1 | Which author/s conducted the interview or focus group? | Page 8 |
| Credentials | 2 | What were the researcher’s credentials? E.g. PhD, MD | Page 28 |
| Occupation | 3 | What was their occupation at the time of the study? | Page 28 |
| Gender | 4 | Was the researcher male or female? | Page 1 and 28 |
| Experience and training | 5 | What experience or training did the researcher have? | Page 8, 12 and 28 |
| *Relationship with participants* |  |  |  |
| Relationship established | 6 | Was a relationship established prior to study commencement? | Page 8 |
| Participant knowledge of the interviewer | 7 | What did the participants know about the researcher? e.g. personal goals, reasons for doing the research | Page 8 |
| Interviewer characteristics | 8 | What characteristics were reported about the interviewer/facilitator? e.g. Bias, assumptions, reasons and interests in the research topic | Page 8 |
| **Domain 2: study design** |  |  |  |
| ***Theoretical framework*** |  |  |  |
| Methodological orientation and Theory | 9 | What methodological orientation was stated to underpin the study? e.g. grounded theory, discourse analysis, ethnography, phenomenology, content analysis | Page 6 |
| *Participant selection* |  |  |  |
| Sampling | 10 | How were participants selected? e.g. purposive, convenience, consecutive, snowball | Page 7 |
| Method of approach | 11 | How were participants approached? e.g. face-to-face, telephone, mail, email | Page 7-8 |
| Sample size | 12 | How many participants were in the study? | Page 9 |
| Non-participation | 13 | How many people refused to participate or dropped out? Reasons? | Page 9 |
| ***Setting*** |  |  |  |
| Setting of data collection | 14 | Where was the data collected? e.g. home, clinic, workplace | Page 8 |
| Presence of non-participants | 15 | Was anyone else present besides the participants and researchers? | No |
| Description of sample | 16 | What are the important characteristics of the sample? e.g. demographic data, date | Page 9-10 |
| ***Data collection*** |  |  |  |
| Interview guide | 17 | Were questions, prompts, guides provided by the authors? Was it pilot tested? | Page 8 |
| Repeat interviews | 18 | Were repeat interviews carried out? If yes, how many? | Page 9 |
| Audio/visual recording | 19 | Did the research use audio or visual recording to collect the data? | Page 8 |
| Field notes | 20 | Were ﬁeld notes made during and/or after the  interview or focus group? | Page 8 |
| Duration | 21 | What was the duration of the interviews or focus group? | Page 8 |
| Data saturation | 22 | Was data saturation discussed? | Page 8 |
| Transcripts returned | 23 | Were transcripts returned to participants for comment and/or correction? | Page 8 |
| **Domain 3: analysis and ﬁndings** |  |  |  |
| ***Data analysis*** |  |  |  |
| Number of data coders | 24 | How many data coders coded the data? | Page 12 |
| Description of the coding tree | 25 | Did authors provide a description of the coding tree? | Page 11 |
| Derivation of themes | 26 | Were themes identiﬁed in advance or derived from the data? | Page 11 |
| Software | 27 | What software, if applicable, was used to manage the data? | Not applied |
| Participant checking | 28 | Did participants provide feedback on the ﬁndings? | Page 8 |
| ***Reporting*** |  |  |  |
| Quotations presented | 29 | Were participant quotations presented to illustrate the themes/ﬁndings? Was each quotation identiﬁed? e.g. participant number | Page 12-21 |
| Data and ﬁndings consistent | 30 | Was there consistency between the data presented and the ﬁndings? | Page 12-21 |
| Clarity of major themes | 31 | Were major themes clearly presented in the ﬁndings? | Page 12-21, 22 |
| Clarity of minor themes | 32 | Is there a description of diverse cases or discussion of minor themes? | No |
