# Supplementary material for: The self-perceived role of tech champions in municipal healthcare services—a descriptive qualitative study
Source: BMC Health Serv Res. 2025 Jul 1;25:856. doi: 10.1186/s12913-025-12994-1 (PMC12220061; doi:10.1186/s12913-025-12994-1)
Supplement: Supplementary file 2 — Additional file 2. [file 12913_2025_12994_MOESM2_ESM.docx]

## Interview Guide- Tech Champions in Municipal Healthcare Services

*Guiding list of topical introductory questions, to be followed by follow-up questions (not listed)*

## Introductory questions

Thank you very much for participating in this interview. First, can you tell us what made you want to be part of an interview about the role of the tech champion?

What professional background do you have?

Can you tell us a little about what you have worked with in the past?

What are the main tasks in your daily work?

What are your thoughts on adopting technology in the municipal health services?

What is required of the staff to succeed with the implementation and use of technology in the health service?

Do you find that there are some technologies that are easier or more difficult than others to implement?

Are there any situations where you think technological solutions are right/wrong?

## The tech champion role (descriptions)

How did you get this role?

Why did you get the role?

How long have you had the role?

What training did you receive for the role?

What do you do as a tech champion?

What would you say if you were to explain what a champion is to others?

## The tech champion role (experiences)

How do you perceive the tech champion’s role?

What skills do the tech champion need to hold?

What is required in the role of champion?

What conditions in the workplace can affect your performance of the champion role?

When did you first hear the term tech champion, and do you think differently about the term now?

Is everyone suitable to be a tech champion?

What competencies does a tech champion need to have? (professional, personal and ethical)

For what reasons are you contacted as a tech champion?

Can you describe how you help others in your department in your role performance?

Who are your supporters in your role performance?

How are your experiences and expertise shared with the other employees and management?

## The Champion Role (Assessments)

What's the best about having this role?

What are the challenges of having this role?

What occupies your mind and time as a tech champion?

Do you have a job description for the tech champion role?

Do you think differently about holding the role now than when you started?

Do you know and collaborate with other tech champions?

What do you think is the advantages and disadvantages for the staff and management having a tech champion in their department?

Do you see any professional and ethical challenges in the role of champion?

What advice would you give to future champions to succeed in your job?

What factors affect the success of a tech champion?

What advice would you give to the management of a municipality that is considering starting with tech champions?

Is there anything the management should think about/be aware of/something that needs to be in place when hiring/appointing a champion? (framework conditions e.g. finances, time, anchoring and organization)

## Final questions (summary)

What do you think is the most important thing convey about tech champion’s role in relation to what we have talked about now?

Is there anything else you would like to add?
